# Relationship between Phenolic Compounds, Antioxidant Properties, and the Allergenic Protein Mal d 1 in Different Selenium-Biofortified Apple Cultivars (*Malus domestica*)

**DOI:** 10.3390/molecules26092647

**Published:** 2021-04-30

**Authors:** Sabrina Groth, Christoph Budke, Timo Weber, Susanne Neugart, Sven Brockmann, Martina Holz, Bao Chau Sawadski, Diemo Daum, Sascha Rohn

**Affiliations:** 1 Hamburg School of Food Science, Institute of Food Chemistry, University of Hamburg, Grindelallee 117, 20146 Hamburg, Germany; sabrina.groth@chemie.uni-hamburg.de (S.G.); svenbrockmann7@gmx.de (S.B.); martina-holz@gmx.de (M.H.); baochau2506@gmail.com (B.C.S.); 2 Department of Plant Nutrition, Osnabrück University of Applied Sciences, 49090 Osnabrück, Germany; c.budke@hs-osnabrueck.de (C.B.); timo.weber23@gmx.de (T.W.); d.daum@hs-osnabrueck.de (D.D.); 3 Department of Crop Sciences, Division Quality and Sensory of Plant Products, Georg-August-Universität Göttingen, 37075 Göttingen, Germany; susanne.neugart@uni-goettingen.de; 4 Department of Food Chemistry and Analysis, Institute of Food Technology and Food Chemistry, Technische Universität Berlin, TIB 4/3-1, Gustav-Meyer-Allee 25, 13355 Berlin, Germany

**Keywords:** apple, biofortification, selenium, antioxidant properties, phenolic compounds, polyphenol oxidase, Mal d 1, allergy

## Abstract

Notable parts of the population in Europe suffer from allergies towards apples. To address this health problem, the analysis of the interactions of relevant allergens with other substances such as phenolic compounds is of particular importance. The aim of this study was to evaluate the correlations between the total phenolic content (TPC), polyphenol oxidase (PPO) activity, antioxidant activity (AOA), and the phenolic compound profile and the content of the allergenic protein Mal d 1 in six apple cultivars. It was found that the PPO activity and the content of individual phenolic compounds had an influence on the Mal d 1 content. With regard to the important constituents, flavan-3-ols and phenolic acids, it was found that apples with a higher content of chlorogenic acid and a low content of procyanidin trimers and/or epicatechin had a lower allergenic potential. This is probably based on the reaction of phenolic compounds (when oxidized by the endogenous PPO) with proteins, thus being able to change the conformation of the (allergenic) proteins, which further corresponds to a loss of antibody recognition. When apples were additionally biofortified with selenium, the composition of the apples, with regard to TPC, phenolic profile, AOA, and PPO, was significantly affected. Consequently, this innovative agronomic practice seems to be promising for reducing the allergenic potential of apples.

## 1. Introduction

Apples contain important compounds that are of health-beneficial relevance. Besides vitamins, a diverse set of minerals and trace elements are present in the fruits [1,2,3,4]. Furthermore, the fruits are rich in secondary plant metabolites, especially flavonoids and phenolic acids [5,6]. In in vitro studies, apple extracts and isolated compounds, especially oligomeric procyanidins, have been shown to influence several mechanisms of cancer development [7]. The consumption of apples is recommended for a healthy diet, as they are hypothesized to reduce the risk of stroke, as well as cardiovascular disease and lung cancer [5,6,8].

However, eating apples can also provoke allergic reactions [9,10,11,12]. Most commonly, there are symptoms that primarily occur in patients with hay fever [13]. About 70% of birch pollen allergy sufferers also show allergic symptoms towards apples because of the chemical-structural homology of the allergenic proteins Bet v 1 and Mal d 1, both belonging to the protein family PR-10 [14]. In a population-based study of young adults (between 20 and 44 years) in 13 European countries, the prevalence of a type I sensitization towards apples ranged from 0% in Iceland to 10.3% in Germany (overall average for all countries: 4.2%) [11]. Type I reaction describes an immediate-type allergy and includes IgE-mediated reactions [15]. Nationwide, approximately four million Germans are impaired by an apple allergy [16,17].

The identification of hypoallergenic apple cultivars is important for dietary recommendations, especially for patients suffering from severe symptoms of apple allergy [18,19]. For this reason, various aspects of apple allergy have been highlighted in numerous scientific studies since the early 1990s [16,17,18,19,20,21,22,23,24,25,26,27,28,29,30,31,32,33,34,35]. The assessment of the allergenic potential of an apple cultivar is a complex issue, as the allergen content of apples is influenced by various factors: the content of allergenic proteins depends primarily on the genotype [17,20,21,22,23,24,25,26] but is also influenced by the level of maturation, postharvest conditions, as well as cultivation conditions and practices, such as the use of selected fertilizers [21,22,24,25,26,27,28,29,36,37].

However, it is hypothesized that certain apple cultivars with comparatively higher total phenolic contents (TPC) are more tolerable with regard to allergenicity. In this context, it is further assumed that the interactions between the polyphenols and the allergenic protein Mal d 1 play an important role in reducing allergenic potential [13,16]. Such interactions can be of a different nature, depending on the structure of the phenolic compounds. Similar to protein–protein interactions, hydrogen, ionic, hydrophobic, and aromatic interactions can occur, leading to a change in the conformation of the proteins [38,39].

Some studies in the literature even describe dependencies on the polyphenol composition, the allergenicity (mainly with regard to Mal d 1), and the activity of polyphenol oxidases, with the latter significantly influencing polyphenol content and composition in apples [16,17,22,28]. Bernert et al. (2012) found a statistically significant correlation between TPC and apple allergenicity [16]. The results of previous studies reported that interactions between oxidized plant polyphenols with allergenic proteins are especially believed to reduce their allergenicity [30,31,40]. During peeling, crushing, or squeezing of the fruits, *p*- and *o*-dihydroxybenzene derivatives are oxidized to quinones, forming soluble and insoluble protein–phenolic complexes with Mal d 1, and thereby “inactivating” the protein [20,30,31,41,42]. Such reactions between polyphenols and proteins can even be between far larger crosslinked melanin-like compounds [39]. A high PPO activity favors the oxidation of phenolic compounds and, consequently, the suppression of the allergenic effect of Mal d 1 [27]. The study described by Kiewning et al. (2013) showed that the activity of PPO seems to be even more important than TPC for lowering the Mal d 1 content. At a high PPO activity, Mal d 1 was reduced, even when the TPC was low [27]. This assumption is supported by the studies described by Kschonsek et al. (2019). They observed that apple cultivars with a high polyphenol content and an equally high PPO activity have a lower allergenicity [20]. Apple cultivars with a high TPC provided a better tolerance. Schmitz-Eiberger et al. (2009) evaluated the relationship between Mal d 1 content, PPO activity, TPC, and antioxidant capacity. The results showed that higher PPO activities and TPCs lead to a diminished extractability of the allergenic proteins [22].

The two phenolic compound classes, phenolic acids and flavonoids, in particular seem to exhibit a high reactivity towards proteins, as many of their chemical structures are highly susceptible to oxidation [39]. These compounds are found in apples, and in some cultivars in particularly high concentrations [43,44,45,46]. Garcia et al. (2007) showed that the addition of the flavan-3-ols catechin and epicatechin can contribute to a reduction of allergenicity. Red-fleshed apple cultivars, which can accumulate phenolic compounds from the anthocyanin class, not only in the fruit peel, but also partially in the fruit flesh, proved to be particularly low in allergenicity [32,33]. Kschonsek et al. (2019) showed that high levels of chlorogenic acid, caffeic acid, and epicatechin were associated with a low concentration of sulfidoleucotrienes, which are synthesized and released by leucocytes after a contact with allergens [17].

As mentioned above, apple composition is influenced by all kinds of physical, chemical, and biological elicitations [47]. In a previous study, the influence of the biofortification of apples with selenium by foliar fertilization was investigated. There, it was found that the content and composition of phenolic compounds were significantly influenced by selenium [48]. In other experiments it was found that even the Mal d 1 content was reduced, in most cases, when the fruits were biofortified with selenate, while apple cultivar and ecophysiological conditions (e.g., climate) were identified as further influencing factors [34].

The aim of the present study was to investigate the relationship between antioxidant properties (as a measure of a phenolic compound’s reactivity), phenolic compound composition, and the allergenic protein Mal d 1, when biofortifying with selenium. Selenium might be an interesting influencing factor in this case, as it is also a redox active trace element. For this purpose, six different apple cultivars from three consecutive growth seasons, and harvested in two different locations, were characterized. With this study, it might be possible to evaluate an innovative agronomic practice for enhancing polyphenols and selenium in apples, while at the same time reducing the content of allergenic proteins.

## 2. Results and Discussion

To analyze the strength and direction of the linear relationship between the Mal d 1 content and the phenolic compounds, as well as the related properties, correlation analyses were performed, and the coefficient of determination was calculated. Furthermore, the influence of a selenium biofortification was also investigated. The contents of the following parameters in the different selenium-biofortified apple samples and controls are shown in Tables S1 and S2 in the Supplementary Materials: selenium content, Mal d 1 content, PPO activity, TPC, content of individual phenolic compounds, and AOA measured by TEAC and ORAC. The biofortification resulted in a significant increase of the selenium content in the fruits, by a factor of 10 to 30 compared to the corresponding controls. Furthermore, the Mal d 1 content of the biofortified apples was reduced in most cases. Regarding the other parameters in the selenium-biofortified apples, a lower variation of PPO activity, higher TPCs upon application of selenite, and changes in the concentration of the major phenolic compounds, such as chlorogenic acid, the fraction of procyanidin trimers, and caffeoylglucoside were observed.

### 2.1. Correlation Analysis between Selenium Content and Mal d 1 Content

The correlation analyses showed no correlation between selenium and Mal d 1 content across all selenium-biofortified apple samples of the six cultivars analyzed. However, a negative correlation was found for most of the individual cultivars, and a high selenium content was therefore associated with a low Mal d 1 content. Variety-specific differences were found. The correlations also varied in strength, with correlation coefficients between 0.0244 and 0.7673 (Table 1). Biofortification with selenium resulted in significantly lower content of the allergenic protein Mal d 1 in ‘Golden Delicious’ and ‘Boskoop’, making these cultivars particularly suitable for a further targeted reduction of the Mal d 1 content by the applied agronomic approach. In the case of ‘Jonagold’, differences between the two cultivation seasons were observed, and a highly significant negative correlation was found for the year 2017. In contrast, a slight positive correlation was determined in the following year. The Mal d 1 content of the cultivars ‘Jonica’ and ‘Elstar’ was only reduced or increased to a small extent by the biofortification. As there was no correlation between Mal d 1 and selenium content in ‘Fiesta’, the Mal d 1 content of this variety was not affected in association to the biofortification approach.

A comparison of the results with the literature can only be made to a limited extent, due to the lack of comparable studies. To the best of our knowledge, the influence of the selenium biofortification of plant foods on allergenic proteins had not been described previously. Nevertheless, a number of other factors have been analyzed for influence on the content of allergenic proteins in apples, including the cultivation system. Schmitz-Eiberger (2011) showed that apples from organic cultivation showed significantly higher Mal d 1 contents [22]. Furthermore, allergic persons showed a higher sensitivity when consuming such apples [29]. The organic cultivation of fruit trees leads to higher susceptibilities towards environmental stress factors such as fungal, bacterial, and viral attack, which were shown to result in a higher biosynthesis rate of Mal d 1 [36]. Mal d 1 is a pathogenesis-related protein, which is synthesized by fruits mainly for defense against such pathogens and occasionally as an response against certain environmental stress conditions [10,12,21]. Therefore, the results of the present study are in line with the previous findings. It is hypothesized that the application of selenium-containing fertilizers leads to the better protection of the fruits against certain stress factors, whereby only a lower synthesis rate of the Mal d 1 protein is required. The induction of further plant-protective substances, such as phenolic compounds, resulting from biofortification with selenium in apples [48] and other crops [49,50,51,52,53,54,55] has also been determined in previous studies. Furthermore, it has already been shown that selenium can protect plants from a range of abiotic stresses such as cold, drought, radiation, salinity, and heavy metals [56,57]. In such cases, it seems that the synthesis of the plant protecting protein Mal d 1 is no longer necessary and therefore reduced in its expression. The role of selenium is associated with the regulation of reactive oxygen species and the stimulation of antioxidant systems [57,58].

### 2.2. Relationship between PPO Activity and Mal d 1 Content

**Figure 1:**
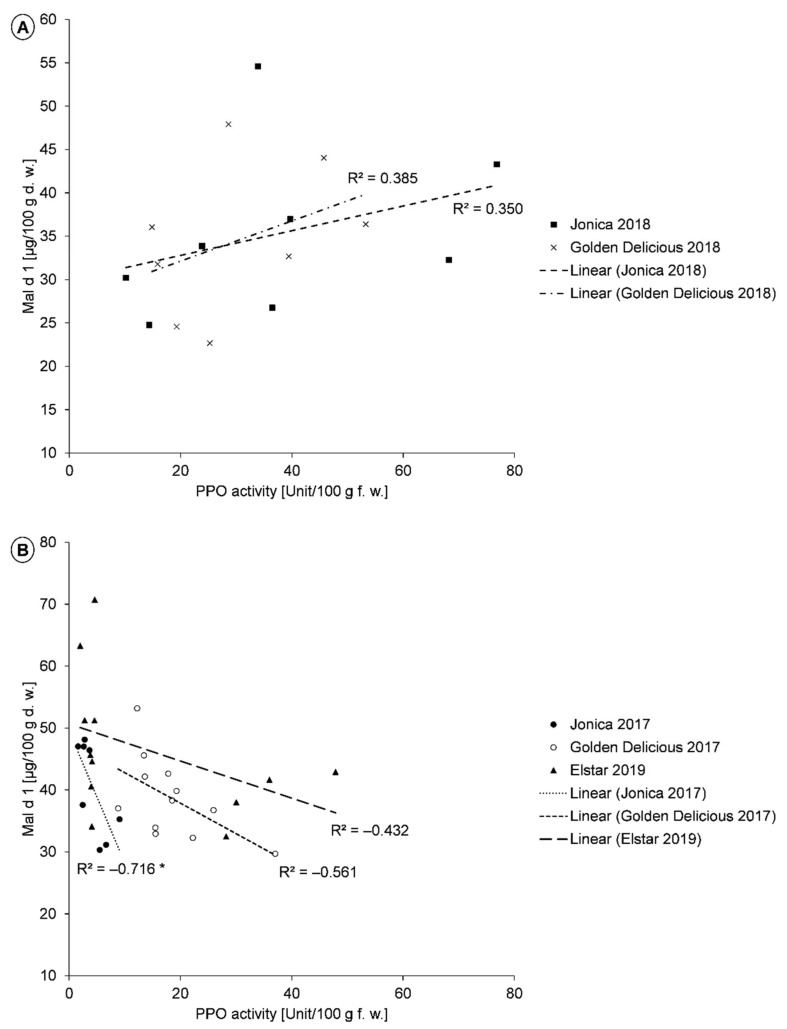
**A**,**B**. Correlation between PPO activity and Mal d 1 content of the cultivars ‘Jonica’ (2018), *n* = 8, ‘Golden Delicious’ (2018), *n* = 12, ‘Jonica’ (2017), *n* = 8, ‘Golden Delicious’ (2017), *n* = 12, and ‘Elstar’ (2019), *n* = 12. The controls and biofortified samples are shown. Indication of the coefficient of determination R^2^ for the respective cultivars. * *p* ≤ 0.05.

The analysis of the correlation between PPO activity and the Mal d 1 content of all investigated samples showed no correlation. There were also no correlations in a separate consideration of the two groups, “controls” and “selenium-biofortified apples” (Table 2). For the analysis of the correlation of the parameters for the individual cultivars, the following was found: for the cultivars ‘Jonica’ and ‘Golden Delicious’ from the cultivation year 2017 and ‘Elstar’ from the year 2019, a low Mal d 1 content was associated with a higher PPO activity (Figure 1A). For ‘Jonica’, the correlation was significant. In contrast, a positive correlation was found for the first two cultivars and for ‘Boskoop’ in the following year 2018 (Figure 1B). There was a high significance for ‘Boskoop’. At this point, the hypothesis was made that the correlation between PPO activity and Mal d 1 content is influenced by genotype, as well as ecophysiological conditions. ‘Fiesta’ and ‘Jonagold’ showed no correlation between PPO activity and Mal d 1 content in all cultivation seasons.

Among others, ‘Jonica’ and ‘Golden Delicious’ were cultivated in 2017 and 2018. Consequently, a comparison between the years of cultivation can be made to analyze the differences in the correlation between PPO activity and the Mal d 1 content. Here, controls and selenium-biofortified samples were included. Both cultivars were found to have a significantly higher PPO activity and lower Mal d 1 content in 2018 compared to the previous year (Table 3). When the data of the individual apples were used for the correlation analysis, an inverse correlation between PPO activity and Mal d 1 content resulted.

In the two years of cultivation, there were different climatic conditions in the apple orchard in Osnabrück, Germany. Compared to the previous year, a significantly higher sunshine duration (+37%) and a significantly lower precipitation (−61%) was recorded for the year 2018 [59].

A negative correlation between PPO activity and Mal d 1 content has been reported for different apple cultivars [20,22,27,32]. In one of their studies, Garcia et al. (2007) investigated the correlation of these parameters in ‘Golden Delicious’ and ‘Jonagold’ and conducted experiments on ‘Golden Delicious’, where an excess of exogenous PPO was added to the apple samples. It was shown that the treatment with PPO reduced allergenicity in the form of a lower IgE-binding capacity of Mal d 1 [32]. Schmitz-Eiberger et al. (2011) also analyzed the relationship between the Mal d 1 content and the PPO activity. Fruits of the three apple cultivars ‘Braeburn’, ‘Topaz’, and ‘Golden Delicious’ were used. The results of that study showed that a higher PPO activity led to a diminished extractability of Mal d 1 [22]. Kiewning et al. (2013) also performed correlation analyses between Mal d 1 content and PPO activity of different cultivars. ‘Elstar’ and ‘Diwa’ showed a high correlation, while the correlation for fruits of ‘Boskoop’ was only moderate [27]. Likewise, Kschonsek et al. found this type of correlation for six different apple cultivars, including ‘Golden Delicious’. Determining the Mal d 1 content and PPO activity after a 60-min oxidation period of the fruits showed a strong decrease of Mal d 1 content, associated with a high PPO activity, as well [20].

A decrease in Mal d 1 content or IgE-binding capacity and the accompanied reduced immunoreactivity seem to result from the reaction of *o*-quinones, deriving from the oxidation of phenolic compounds, with the proteins. As PPO catalyzes this reaction, a high enzyme activity leads, accordingly, to high *o*-quinone contents. These in turn can lead to an irreversible change in the tertiary structure of the allergen by modifying the nucleophilic amino acid side chains of the proteins, with the possibility of follow-up polymerizations [60]. Due to these cross-linkages, conformational epitopes of the allergen get lost, which reduces or even eliminates allergenicity [32,41,61].

To investigate the influence of selenium biofortification on allergenicity, correlation analyses of the individual cultivars were performed for the controls and the biofortified samples (Table 2). No consistent effects were found across all cultivars. For ‘Fiesta’ and ‘Golden Delicious’ from the year 2017, and ‘Jonagold’ from the year 2018, the biofortification led to a change in correlation towards negative values. For ‘Jonagold’ (2017) and ‘Jonica’ (2018), a change towards a positive correlation was observed for the selenium-biofortified samples. ‘Golden Delicious’ from the year 2018 and ‘Elstar’ from the year 2019 showed a stronger negative correlation for the biofortified samples compared to the controls. The correlation between PPO activity and Mal d 1 content was only significant for ‘Jonagold’.

### 2.3. Analysis of the Relation between TPC and Mal d 1 Content

**Figure 2:**
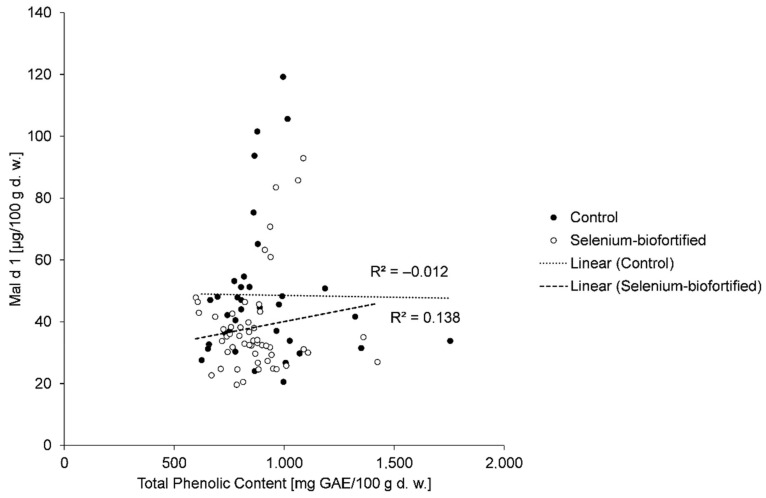
Correlation between TPC and Mal d 1 of all control samples (*n* = 36) and of all selenium-biofortified samples (*n* = 52).

The analysis of the correlation between TPC and Mal d 1 showed no correlation, when considering all samples, and comparing “control” and “selenium biofortification” (Figure 2). A separate analysis of the individual cultivars showed only a weak negative correlation for ‘Jonica’ (2017) and ‘Jonagold’ (2018), and only a weak positive correlation for ‘Golden Delicious’ (2018) and ‘Elstar’ (2019) (Table 2). No correlation was of statistical significance. At this point, no trend was identified. It was therefore assumed that TPC alone does not, or only to a small extent, influence the content of allergenic proteins.

In line with this, Kiewning et al. (2013) and Kschonsek et al. (2019b) concluded that TPC plays only a minor role with regard to Mal d 1 content. In contrast, the activity of PPO proved to be more important for the reduction of Mal d 1. At high PPO activity, Mal d 1 activity can be reduced, even when the TPC is low [20,21,22,23,24,25,26,27].

According to the consistent results of several studies, there is an inverse relationship between TPC and the allergenicity of apples [17,22]. Bernert et al. (2012) analyzed the cultivars ‘Red Boskoop’ and ‘Golden Delicious’, among others, and found that apple cultivars with a high content of total polyphenols provided a better tolerance for apple allergy sufferers [16]. Kschonsek et al. (2019a) detected an inverse correlation between high TPC and low in vitro allergenicity of apples [17]. One of the first attempts to evaluate the relationship between Mal d 1 content and PPO, TPC, and antioxidant capacity in different apple cultivars was reported by Schmitz-Eiberger et al. (2011). Their results showed that higher PPO activity and TPC lead to a diminished extractability of the allergenic protein Mal d 1 [22]. It is assumed that oxidative reactions between apple polyphenols and the allergen are responsible [30,31]. The reduction in allergenicity could be due to the masking of IgE-binding sites on the allergenic protein, through cross-linking of proteins induced by oxidative enzymes [39,41]. PPO is the main factor involved in these oxidative reactions in fruit [32]. A decrease in the allergenic potential of the protein Pru av 1 in the presence of polyphenols and PPO was also observed in cherries [61].

The biofortification of apples with selenium did not result in any consistent effects across cultivars with regard to the relationship between TPC and PPO activity. For example, a change in correlation from negative values (as estimated for the controls) to a positive correlation was observed in the biofortified samples of the cultivar ‘Fiesta’ from the year 2017. This effect also occurred for ‘Jonica’ (2018). ‘Golden Delicious’ from the year 2017 showed a significantly higher negative correlation, while all other cultivars showed only marginal differences in correlation between the two parameters.

### 2.4. Individual Phenolic Compounds Influence the Content of Mal d 1

A qualitative and quantitative analysis of the phenolic compounds of the apple samples from the cultivation year 2017 was performed by HPLC-MS^n^. The following compounds were detected: the dihydrochalcones phloretin-2-xylosyl-glucoside and phloretin-2-glucoside, the flavan-3-ol epicatechin, a procyanidin dimer and a fraction of procyanidin trimers, the hydroxycinnamic acid derivatives caffeoylglucoside and chlorogenic acid, as well as the flavonols quercetin-3-*O*-galactoside, quercetin-3-*O*-xyloside, and quercetin-3-*O*-glucoside.

The main compounds in apples are chlorogenic acid, the sum of the quercetin glycosides, the sum of the two phloretin glucosides, and epicatechin. Significant differences were found between the cultivars, especially in the content of chlorogenic acid and quercetin glycosides (Table S2, Supplementary Materials).

The cultivar ‘Fiesta’ was characterized above all by a high proportion of chlorogenic acid (40%). The other cultivars only had proportions of 21–27%. Furthermore, differences appeared in the proportion of epicatechin: ‘Fiesta’ contained an average of 15%, while the others had only 9–10%. With regard to the phloretin glucosides and the quercetin glycosides, ‘Fiesta’ contained significantly less of these, at 8% and 23%, compared with 12–14% and 28–41% for the other cultivars, respectively.

Kschonsek et al. (2018) also reported high levels of chlorogenic acid in various apple cultivars. For this purpose, they analyzed the old cultivars ‘Ontario’ and ‘Dülmener Rosenapfel’ and the comparatively newer cultivars ‘Braeburn’ and ‘Granny Smith’ and found significant differences between the old and the new cultivars. Regarding the profile of phenolic compounds, chlorogenic acid was the main polyphenol in the old apple cultivars with a percentage of around 63%. The new apple cultivars ‘Braeburn’ and ‘Granny Smith’, on the other hand, contained a significantly lower proportion of chlorogenic acid, amounting for 15.4% [2].

In the present study, correlation analyses were performed for the main individual phenolic compounds and Mal d 1 content (Table 3). Across all samples, without considering cultivar or biofortification, the correlation coefficient between Mal d 1 content and the individual phenolic compounds was highest for the fraction of procyanidin trimers, followed by the caffeoylglycosides. For the more complex procyanidins, there was a positive correlation, with a high significance; samples with a higher content of procyanidin trimers also had a higher content of Mal d 1. In contrast, there was an inverse correlation for caffeoylglycosides and Mal d 1.

**Figure 3:**
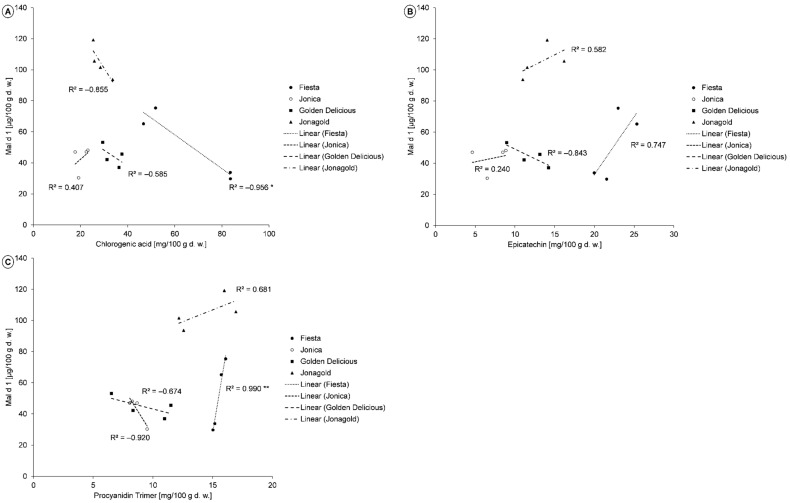
**A–C**. Correlation between the individual phenolic compounds and the Mal d 1 content in apple samples of the cultivars ‘Fiesta’, ‘Jonica’, ‘Golden Delicious’, and ‘Jonagold’, *n* = 4 for each variety. (**A**) Chlorogenic acid; (**B**) epicatechin; (**C**) procyanidin trimer. * *p* ≤ 0.05; ** *p* ≤ 0.01.

A separate analysis of the correlation between the individual phenolic compounds and the allergenic potential for the controls of each cultivar showed different relationships, depending on the cultivar. For chlorogenic acid, a negative correlation was found for ‘Fiesta’, ‘Golden Delicious’, and ‘Jonagold’ (Figure 3A). High levels of epicatechin were observed in association with high Mal d 1 levels for ‘Fiesta’ and ‘Jonagold’, whereas there was a negative correlation for ‘Golden Delicious’ (Figure 3B). Regarding the fraction of procyanidin trimers, a positive correlation was observed for ‘Fiesta’ and ‘Jonagold’ and a negative correlation for ‘Jonica’ and ‘Golden Delicious’ (Figure 3C). The correlation coefficients of caffeoylglucosides and Mal d 1 were low (−0.35 ≥ R^2^ ≤ 0.06), except for ‘Fiesta’. Therefore, the content of this phenolic compound probably plays only a minor role with regard to the allergenic potential. The sum of phloretin glucosides correlated positively with the Mal d 1 content in ‘Jonica’ and ‘Jonagold’. Furthermore, a negative correlation was observed between the sum of quercetin glycosides and the Mal d 1 content in all cultivars, except ‘Jonagold’.

The correlation between individual phenolic compounds and the Mal d 1 content of numerous cultivars has already been determined and described in the literature [16,17,20,27,35]. Kiewning et al. (2013) analyzed the abovementioned parameters for the cultivars ‘Elstar’, ‘Diwa’, and ‘Boskoop’ and found a low to moderate correlation between catechin, as well as epicatechin, and Mal d 1 content. In contrast to ‘Elstar’ and ‘Boskoop’, the correlation between Mal d 1 and catechin, as well as epicatechin, of the cultivar ‘Diwa’ was negative [27]. Moreover, in the present study, low to moderate correlation coefficients were found with regard to epicatechin, as well as different dependencies on cultivar.

Bernert et al. (2012) performed an analysis of the correlation between the content of phenolic compounds and the apple allergy tolerance for different cultivars, including ‘Golden Delicious’. They identified chlorogenic acid as the main polyphenol in all apple cultivars tested. A statistical evaluation showed a negative correlation between the chlorogenic acid content and the tolerance claims. When apples contained high levels of chlorogenic acid, they were better tolerated by allergy sufferers [16]. The present study confirmed this relationship to a large extent, since in most varieties a high chlorogenic acid content was correlated with a low content of Mal d 1. Due to this, a better tolerance is assumed.

Kschonsek et al. (2019b) conducted experiments on the influence of enzymatic browning with regard to in vitro allergenicity in two old and two new apple cultivars and drew conclusions on the relationship between phenolic compounds and allergenic potential. A more intense enzymatic browning occurred in the cultivar ‘Ontario’ compared to ‘Dülmener Rosenapfel’. At the same time, a 25% higher decrease in TPC was observed for ‘Ontario’. This may have been due to the higher content of total flavanols (50%) and total hydroxycinnamic acids (15%), as the phenolic compound classes are very good substrates for PPO [20,27,62]. The higher degree of browning was associated with a lower in vitro allergenicity. Correlation analyses showed that high levels of chlorogenic acid, caffeic acid, and epicatechin were associated with the lower in vitro allergenicity of the apples [20]. The present study could only partially confirm these results. Thus, a negative correlation between chlorogenic acid and Mal d 1, which is directly related to allergenicity, was also found in three of the four varieties analyzed. Caffeic acid was not identified in the apple samples. In comparison to the study by Kschonsek et al., high levels of epicatechin associated with low Mal d 1 contents were observed only in Golden Delicious [20]. In contrast, the varieties Fiesta, Jonica, and Jonagold showed a positive correlation of these two parameters. These differences can be explained by the different cultivars.

In a recent study by Romer et al. (2020), the correlation between the phenolic profile and Mal d 1 content was investigated in 16 different apple cultivars. No correlation with the allergen content was found with regard to the levels of flavonols, anthocyanins, and phenolic acids. The flavan-3-ols catechin and epicatechin, as well as the procyanidins B1, B3, and a non-specified procyanidin, showed a high positive correlation with the allergen content [35]. As already explained, the present study was able to confirm the positive correlation between the content of epicatechin and Mal d 1. Variety-specific differences were present with regard to procyanidins. A positive correlation was also observed for ‘Fiesta’ and ‘Jonagold’, whereas a low procyanidin content was correlated with a low Mal d 1 content in ‘Golden Delicious’ and ‘Jonagold’.

The allergenicity of apples seems to be mostly influenced by the procyanidins, as well as their monomer epicatechin, and chlorogenic acid. However, there are differences between the varieties. Here, a low procyanidin and epicatechin content, as well as a high content of chlorogenic acid, had an enhanced effect on Mal d 1 content, since low levels of the allergen were present here. With regard to cultivars being generally low in allergens, cultivars with a low procyanidin and epicatechin content and a high chlorogenic acid content seem to, therefore, be advantageous. As only very low correlation coefficients were measured between the other phenolic compounds and Mal d 1, the content of these substances probably had no influence on the overall allergenic potential of the apples.

**Figure 4:**
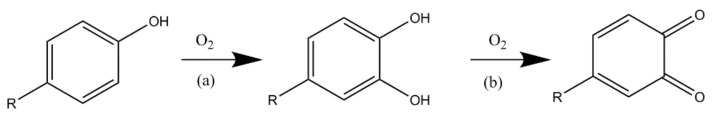
Reactions of PPO as (**a**) monophenolase: in the presence of oxygen, the hydroxylation of phenol derivatives to catechols is catalyzed. (**b**) *o*-Diphenolase activity: the catechols are oxidized to *o*-quinones by the activity of PPO.

In most cases, biofortification resulted in lower procyanidin and epicatechin contents and higher levels of chlorogenic acid associated with a lower Mal d 1 content. Therefore, this agronomic practice seems to be suitable for the reduction of allergenic potential. Polyphenols, and especially their oxidation products, quinones, are among the most reactive ingredients in apples. The reaction of phenolic compounds, as phenoxy radicals, quinones, or semiquinone radicals, results in irreversible interactions with proteins [38,39]. The oxidative degradation of phenolic compounds catalyzed by PPO leads to the formation of *o*-quinones (Figure 4).

The *o*-quinones are very reactive, they can subsequently form dimers/oligomers/polymers with other phenolic compounds (brown colored melanins), as well as adducts with proteins. The oligomers, in turn, can be re-oxidized and covalently crosslink proteins [38,39].

The potential anti-allergenic properties of the phenolic compounds are based on different molecular mechanisms: On the one hand, the tertiary structure of the proteins can be altered to produce a lack of antibody recognition. This can be caused either by the polyphenols themselves, their oxidized forms (*o*-quinones), or even more directly by PPO. First of all, polyphenols can act as ligands for the hydrophobic cavity [35,63,64]. Due to structural similarity, PPO can use the phenolic amino acid tyrosine as a substrate, in addition to other phenolic compounds. When tyrosine is in the protein structure of the allergens oxidized, there can be a formation of covalent crosslinks within the protein(s) and, consequently, a conformational change and a loss of antibody recognition [20,35,65]. Another mechanism concerns the influence of phenolic compounds on mast cells and the prevention of histamine secretion [27,35,42,66]. Thus, polyphenols are able to influence the binding between IgE antibodies and the FCεRI receptor on the mast cell surfaces [23,24,25,26,27,28,29,30,31,32,33,34,35,36,37,38,39,40,41,42,43,44,45,46,47,48,49,50,51,52,53,54,55,56,57,58,59,60,61,62,63,64,65,66,67], resulting in a lower amount of released histamine and, thus, in a lower allergic recruitment [35].

Furthermore, interactions between the phenolic compounds and the allergenic proteins are possible, which can influence digestion in the gastrointestinal tract and, thus, inactivating the allergenic effect. Thus, protein–phenol adducts can be formed, which are enzymatically less digestible [38,39]. As already described above, irreversible bonds between phenolic compounds and proteins can be formed, whereby phenolic compounds are oxidized into quinones, which in turn can react with nucleophilic groups of the protein molecule. These interactions can affect the structure, functionality, and quality of the proteins, while bioavailability can also be affected by reduced digestibility in the gastrointestinal tract [68,69].

### 2.5. Relationship between AOA and Mal d 1

AOA is a measure for the reactivity of phenolic compounds. In the present study, it was determined using the two well-known assays TEAC and ORAC, which are based on different reaction mechanisms and, thus, allow a broader measurement of the AOA and reactivity, respectively. With regard to the determination of AOA in phenol-rich samples such as apples, the TEAC approach is well established. The stable ABTS-^+^ radical used here reacts rapidly with antioxidants and many phenolic compounds with low redox potential. When using the ORAC assay, AOA can be measured over a longer period via the antioxidant inhibition being induced by exogenous peroxyl radicals, and representing a biologically relevant mechanism. The potential effects of secondary antioxidant compounds can also be measured and underestimation can be prevented [70].

**Figure 5:**
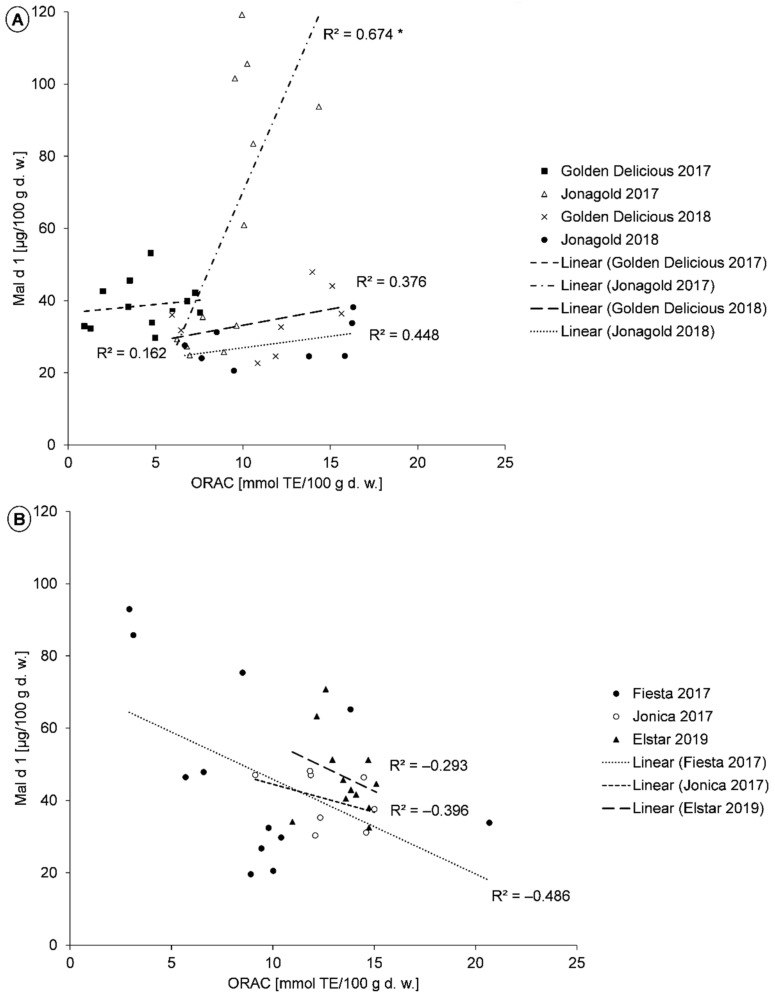
**A**,**B**. Correlation between ORAC-values and Mal d 1 contents in the apple fruits of the cultivars (**A**) ‘Golden Delicious’ and ‘Jonagold’, harvested in 2017 (*n* = 12 for each variety) and 2018 (*n* = 8 for each variety) *n* Osnabrück; (**B**) ‘Fiesta’ (*n* = 12), and ‘Jonica’ (*n* = 8), harvested 2017 in Osnabrück and ‘Elstar’ (*n* = 12), harvested 2019 in Jork. * *p* ≤ 0.05.

Correlation analyses between the AOA measured with the TEAC assay and the Mal d 1 content showed no correlation for all samples, and for the selenium-biofortified samples, in particular (Table 2). However, the controls showed an inverse correlation, with the AOA being higher at low Mal d 1 levels. The analysis of the individual cultivars showed a positive correlation for ‘Golden Delicious’ from the year 2018 and ‘Elstar’. For all other cultivars, an inverse correlation of varying degree was observed (Table 2). Furthermore, correlation analyses were performed between the ORAC value and the Mal d 1 content. However, the correlation between AOA and Mal d 1 content was weakly negative in all samples, as well as in all controls and in all selenium-biofortified samples (Table 2). ‘Golden Delicious’ and ‘Jonagold’ showed a positive correlation between ORAC values and Mal d 1 contents in both years of cultivation (Figure 5A), whereas a negative relation was present for ‘Fiesta’, ‘Jonica’, and ‘Elstar’ (Figure 5B). Only in the case of ‘Jonagold’ rom 2017 was the correlation of statistical significance.

In addition to the TPC and the PPO activity, AOA can be assigned to a certain role in apple allergenicity [22,27,31,32,40,71,72]. Garcia et al. (2007) and Schmitz-Eiberger et al. (2011) investigated in their studies the relationship between AOA and allergenicity in ‘Golden Delicious’ apples. They found that AOA and allergenicity were positively correlated [22,32]. This correlation was only partially observed in the present study. Based on the data available, it is clear that a positive correlation is only valid for individual cultivars. This applies for example to ‘Golden Delicious’ and ‘Jonagold’.

Garcia et al. (2007) treated apples of the cultivar ‘Golden Delicious’ with the synthetic antioxidant dietyldithiocarbamic acid (DIECA). DIECA was added to the samples in sodium phosphate buffer or in succinate-lactate buffer and incubated for 5 to 24 h. A significant inhibition of the IgE-binding of Mal d 1 was found to result from an inhibition of the complex reaction between oxidized phenolic compounds and Mal d 1. The Mal d 1 content in the samples treated with DIECA was higher than the controls. Compared to the controls, the IgE-binding of Mal d 1 in the DIECA-treated samples did not decrease as much, due to a parallel inhibition of further endogenous enzymes [32].

Schmitz-Eiberger et al. (2011) determined the antioxidant capacity of the three apple cultivars ‘Braeburn’, ‘Topaz’, and ‘Golden Delicious’. Unfortunately, they did not specify the method or specific values for AOA in their publication. Regarding the relationship between the AOA, the PPO activity, and the Mal d 1 content, it was found that the Mal d 1 content and the AOA were lowest and the PPO activity was highest in ‘Braeburn’. For ‘Golden Delicious’, the three parameters were in a medium range. For ‘Topaz’, a high TPC, a high catechin content, a relatively low PPO activity, and a high AOA were measured. Schmitz-Eiberger et al. (2011) assumed that the IgE-binding of Mal d 1 was reduced by the low progression of oxidative processes (low PPO activity) or by the inhibition of these processes resulting from a high AOA. The authors found that a higher PPO activity and TPC resulted in a diminished extraction of the protein Mal d 1, whereas higher AOA inhibited the interactions between oxidized phenolic compounds and Mal d 1. This results in a higher allergenicity and a “normal” extractability of Mal d 1 [22,72].

With regard to AOA measured by TEAC, different changes were found between the respective controls and biofortified samples. Thus, a consistent trend by biofortification with selenium was excluded. The evaluation of the correlations between the ORAC value and the Mal d 1 content, depending on the selenium biofortification, showed a trend across several cultivars. A positive correlation was found for the biofortified samples of ‘Jonica’, ‘Golden Delicious’, and ‘Jonagold’ from the year 2017, and for ‘Boskoop’, ‘Jonica’, and ‘Jonagold’ from the year 2018. However, the correlation was negative in the corresponding controls.

## 3. Materials and Methods

### 3.1. Chemicals

Disodium hydrogen phosphate dodecahydrate was purchased from Bernd Kraft GmbH (Duisburg, Germany). Sodium dihydrogen phosphate monohydrate and 3,3’,5,5’-tertamethylbenzidine were from AppliChem GmbH (Darmstadt, Germany). Catechol was from ThermoFisher GmbH (Kandel, Germany). Aceton and ethanol were purchased from VWR International LLC (Fontenay-sous-Bois, France). Bovine serum albumin (BSA), citric acid monohydrate, hydrochloric acid (25%), hydrogen peroxide (30%), sodium chloride, and Tween^®^ 20 were purchased from Carl Roth GmbH & Co. KG (Karlsruhe, Germany). Potassium dihydrogen phosphate, sodium carbonate, and sulphuric acid were purchased from Grüssing GmbH (Filsum, Germany) and potassium peroxodisulphate was from Fisher Scientific UK Ltd. (Loughborough, UK). Folin-Ciocalteu’s phenol reagent, nitric acid (65%), polyvinylpyrrolidone, potassium dihydrogen phosphate, and sodium diethyldithiocarbamat were from Merck KgaA (Darmstadt, Germany). Galllic acid and 2,2ʹ-azobis(2-methylpropionamidine) dihydrochloride (AAPH) were from Fisher Scientific GmbH (Schwerte, Germany). 2,2ʹ-Azino-bis-(3-ethylbenzthiazoline−6-sulfonic acid) diammonium salt (ABTS), trolox, and fluorescein were purchased from Sigma-Aldrich Chemie GmbH (Deisenhofen, Germany). All of the chemicals were of analytical grade. Water was purified using a Milli-Q water system (PURELAB^®^, Elga LabWater, Veolia Water Technologies GmbH, Celle, Germany) and used for buffers, extraction solvents, and for the dilution of sample extracts.

### 3.2. Sample Material

For the analysis of the relationship between antioxidant properties, phenolic compounds, and the allergenic protein Mal d 1, six different apple cultivars, grown in three subsequent years in two different locations, were characterized. Apples of the cultivars ‘Fiesta’, ‘Golden Delicious’, ‘Jonagold’, and ‘Jonica’ were cultivated in 2017 at the Horticultural Research Station of the Osnabrück University of Applied Sciences, Germany (52°31′06.5″N 8°02′84.4″E; 69 m a.s.l.). In the following year, the cultivars ‘Boskoop’, ‘Golden Delicious’, Jonagold’, and ‘Jonica’’ were cultivated in Osnabrück as well. In 2019, apples of the cultivar ‘Elstar’ were cultivated in an orchard of a commercial fruit farm in the “Alte Land” region, Jork, Germany (53°30′37.4″N 9°44′44.6″E; 4 m a.s.l.). The location conditions in Osnabrück and Jork and the design of the field experiments have been already described [34,48]. The apple trees were biofortified with a total of 0.075–0.450 kg selenium per hectare and at a meter canopy height (Se/ha x m CH) by applying foliar sprays. Apples of the cultivar ‘Fiesta’ were sprayed once every two weeks before the harvest in 2017. All other cultivars were treated repeatedly (2–7 times) between mid-June and the end of September. The last application always took place at least two weeks before harvest.

The detailed composition of the selenium-containing fertilizers used and the equipment for application have already been described by Groth et al. [34]. The selenium content was determined in air-dried, ground material of fresh apple samples, while the activity of the polyphenoloxidase was measured in frozen and thawed samples. All other parameters were determined in lyophilized apples. Freeze-drying was performed after homogenization, as described in Groth et al. [48]. For the determination of the selenium content, a sample set of ten randomly chosen apples per treatment and repetition was analyzed. For the determination of the other parameters, a sample set of four randomly chosen apples per treatment and repetition was analyzed.

### 3.3. Determination of the Polyphenol Oxidase (PPO) Activity

PPO activity was determined as described by Groth et al. [48]. About 10 g of the frozen sample was weighed, crushed in a mortar, and mixed with 25 mL of a phosphate buffer (0.05 M, pH 7.0). The subsequent incubation time was 120 min at 4 °C, in the dark. The supernatant obtained after centrifugation (15 min, 4 °C, 3225 g) was used for photometric measurement in a 96-well microtiter plate. First, 30 µL of the sample extract was pipetted into a well and either 270 µL of a phosphate buffer (0.2 M, pH 5.5) as blank sample or 270 µL of a catechol solution as appositive control (0.1 M in 0.2 M phosphate buffer, pH 5.5) was added. Measurement over a period of 10 min was performed at a wavelength of λ = 420 nm at 25 °C with a microplate reader (BioTek Synergy HT, BioTek Instruments Inc., Winooski, VT, USA). The change in absorbance was recorded every 60 s. The enzyme activity of the samples was expressed as activity units per 100 g of fresh weight (f.w.), where one unit was defined as the change of 0.01 in the absorbance value per minute [48].

### 3.4. Method for Extracting Phenolic Compounds

The phenolic compounds were extracted from the apple samples using the method according to Groth et al. [48]. For this, 60 mg of the lyophilized sample was mixed with 1 mL of extraction solvent (50% aqueous acetone and 0.1% HCl (*v/v*)) and treated in an ultrasonic bath (5 min, 30 °C). Four glass beads (i. d. 4 ± 0.3 mm) were added and the sample was ground and mixed in a ball mill (5 min, 25 Hz) (RETSCH^®^ MM 400, Retsch GmbH, Haan, Germany) and then centrifuged (5 min, 20,817 g). Three treatments were carried out with the ball mill. The supernatants were combined and filled up to a volume of 4 mL [48].

### 3.5. Determination of the Total Phenolic Content (TPC) according to Folin-Ciocalteu

The TPC was evaluated using a modified Folin-Ciocalteu method [48]. Twenty microliters of the sample extract was mixed with 100 µL Folin-Ciocalteu phenol reagent (1:10; *v/v*) and 80 µL of an aqueous 7.5% (*w/v*) sodium carbonate solution in a 96-well microtiter plate and incubated in the dark for 2 h. The photometric determination of TPC was performed at a wavelength of λ = 765 nm with a microplate reader (BioTek Synergy HT). TPC values are given in gallic acid equivalents per 100 g of dry weight (mg GAE/100 g d.w.) [48].

### 3.6. Identification and Quantification of Single Phenolic Compounds Using High Performance Liquid Chromatography Mass Spectrometry (HPLC-MS)

Extraction of phenolic compounds from the lyophilized apple samples was carried out with 60% aqueous methanol in a triple extraction, according to Groth et al. (2020a) and Neugart et al. (2017) [48,73]. Phenolic compound identification and quantification were determined using an 1100 series HPLC system (Agilent Technologies GmbH, Waldbronn, Germany) equipped with an Ascentis^®^ Express F5 column (150 mm × 4.6 mm, 5 μm, Sigma-Aldrich Chemical Co., St. Louis, MO, USA) and a photodiode array detector. Eluent A was 0.5% acetic acid, and eluent B was 100% acetonitrile, used in a gradient modus. The wavelengths of 280 nm, 320 nm, and 370 nm were used for the determination of phloretin glycosides and flavan−3-ols, hydroxycinnamic acid derivatives, and non-acylated flavonol glycosides, respectively. The hydroxycinnamic acid derivatives and flavonoid glycosides (chlorogenic acid, catechin, epicatechin, phloretin-2-*O*-glucoside, and quercetin-3-*O*-glucoside) were identified as deprotonated molecular ions and characteristic mass fragment ions, according to Schmidt et al. (2010) [74], by HPLC-DAD-ESI-MS^n^ with an Agilent ion trap mass spectrometer (Agilent Technologies Deutschland GmbH, Waldbronn, Germany) in negative ionization mode. The results are presented as mg/100 g dry weight [48].

### 3.7. Analysis of the Antioxidant Activity (AOA) Using the Trolox Equivalent Antioxidant Capacity Assay (TEAC) and the Oxygen Radical Absorbance Capacity Assays (ORAC)

AOA was determined by TEAC and ORAC assays. As both are based on different reaction mechanisms, more information about the AOA and reactivity of the phenolic compounds can be made than when using only one assay. The measurements were performed as described by Groth et al. [48]. For determining TEAC, a solution was first prepared with the ABTS^+^ radical and diluted with a phosphate buffer (75 mM, pH 7.4) for reaching an absorbance of E_730_ = 0.700 ± 0.050 (ABTS working solution II). Twenty microliters of various dilutions of the samples, trolox for calibration, or water (blank value) was applied in a 96-well microtiter plate, and then 200 μL of ABTS working solution II was added. The absorption was measured after 6 min incubation at 30 °C and a wavelength of λ = 730 nm with the BioTek Syngergy HT microplate reader. AOA was calculated as trolox equivalent per 100 g dry weight (mmol TE/100 g d.w.) [48].

For the determination of the ORAC, 10 µL of each sample, trolox, or water was applied in a 96-well microtiter plate. Thirty-five microliters of a fluorescein solution (1.2 µM) was added. Subsequently, 100 μL phosphate buffer, or 250 μL in the case of the negative control, was added. After a 10 min-incubation period at 37 °C in the BioTek Synergy HT microplate reader, 150 μL of an AAPH solution (c = 129 mM) was added to the blank value, standards, and samples. The measurement, which is based on fluorescence quenching, was performed at 37 °C, an excitation wavelength of λ = 485 nm, and an emission wavelength of λ = 528 nm. The course of the reaction was recorded for 120 min, with one measurement every two minutes. AOA was also calculated as trolox equivalent per 100 g dry weight (mmol TE/100 g d.w.) [48].

### 3.8. Extraction of Proteins

For the determination of the Mal d 1 content, proteins were first extracted from the lyophilized apple samples using the method described by Groth et al. [34]. For this purpose, 1.0 g was weighed into grinding bowls and 15 mL of a Björksten extraction buffer with some modifications was added [40]. The addition of sodium azide was omitted, due to its inhibitory effect on the polyclonal, HRP-labeled goat anti-mouse antibody used for the measurement of the Mal d 1 content by direct ELISA (MERCK KGAA, 2019). Extraction was performed with a ball mill for 10 min at 25 Hz (RETSCH^®^ MM 400). Then, samples were transferred into 15 mL-tubes and subsequent centrifugation was performed (10 min, 20,817× *g*). The supernatant was transferred to another 15 mL-tube and extraction was repeated twice more using the ball mill. All supernatants were combined, concentrated to a volume of 3–4 mL by a gaseous stream of nitrogen, and filled up to 5 mL in a volumetric flask with the phosphate buffer solution [34].

### 3.9. Determination of the Mal d 1 Content Using ELISA

The use of a direct ELISA for the determination of the Mal d 1 content has already been described by Groth et al. [34]. First, a 1:10 dilution of the sample extracts was prepared and 10 µL thereof was pipetted into a 96-well microtiter plate. These were further diluted by adding 190 µL of a Björksten extraction buffer and incubated for 22 h at 4 °C. For calibration, 200 µL of a recombinant, commercially available Mal d 1 solution (2 µg/mL, Biomay AG, Vienna, Austria) was added to each well. A calibration series from 0.1 to 2.0 µL/mL was prepared. After incubation, washing was repeated five times with 300 µL PBS-T buffer each time (PBS buffer: sodium chloride 0.034 mmol/L, potassium hydrogen phosphate 0.016 mmol/L; pH 7.0; + 0.5% Tween 20). A 1% BSA solution was added as blocking reagent and incubated for 2 h at room temperature. Washing was then done five times with 300 µL PBS-T solution each. Then, 200 µL of a HRP-labelled goat anti-mouse antibody (goat anti-mouse IgG antibody, peroxidase conjugated, H+L, Merck KGaA, Darmstadt, Germany) [75] was added and incubated for 18 h at 4 °C. For the preparation of the reaction solution, 10 mL of a citric acid buffer (6.327 g/L citric acid monohydrate in bidest. water, pH 4.1) was mixed with 0.5 mL of a TMB reagent (2.410 g/L 3,3’,5,5’-tertamethylbenzidine, 0.5 mL hydrogen peroxide (30%), 100 mL acetone, and 900 mL ethanol) [76]. Two-hundred microliters were pipetted into every well and incubated for 90 min at room temperature in the dark. As stop solution, 50 µL sulphuric acid (2 M), was added and the photometric measurement was performed at λ = 450 nm at 30 °C in a microplate reader (BioTek Synergy HT). The Mal d 1 content was given in mg/100 g f.w. [34].

### 3.10. Statistical Analysis

The number of analyses per application with selenium fertilizer or control was *n* = 2. All of the analyses were repeated twice. The data in the Supplementary Materials, Table 1 and Table 2, are given in mean ± standard deviation and were further evaluated using Microsoft Excel (Microsoft Office Professional Plus 2016, Redmond, WA, USA). To test the correlation between the individual parameters, correlation analyses were performed, also using Microsoft Excel, and the coefficient of determination R^2^ was determined.

## 4. Conclusions

Several influencing factors have been identified regarding the allergenicity of apples with Mal d 1 content as a measure. In particular, PPO activity and the content of individual phenolic compounds, such as chlorogenic acid, epicatechin, and the fraction of procyanidin trimers, were related to the Mal d 1 content. Biofortification of apples with selenium seems to be promising as an agronomic practice for reducing the allergenic potential of apples. The molecular mechanisms are mainly based in the phenol–protein interactions, where the *o*-quinones resulting from oxidation by PPO lead to an irreversible conformational change of the allergens. As a result, the conformal epitopes of the allergen are affected and allergenicity is reduced. Consequently, it seems to be valuable to take apples into account that already have a low content of allergenic proteins, to biofortify them with selenium, and stimulate TPC formation in this way.

## Figures and Tables

**Table 1:** Correlation between Selenium Content and Mal d 1 Content.

Cultivar and Year of Cultivation	Correlation Coefficient Selenium—Mal d 1
All	−0.0154
‘Fiesta’ 2017	0.0244
‘Jonica’ 2017	−0.4099
‘Golden Delicious’ 2017	−0.6493 *
‘Jonagold’ 2017	−0.7673 **
‘Boskoop’ 2018	−0.7463 *
‘Jonica’ 2018	−0.3524
‘Golden Delicious’ 2018	−0.7318 *
‘Jonagold’ 2018	0.2491
‘Elstar’ 2019	0.3922

* *p* ≤ 0.05; ** *p* ≤ 0.01.

**Table 2:** Relationship between PPO Activity, TPC, Antioxidant Activity (TEAC and ORAC), and Mal d 1 Content.

Cultivar and Year of Cultivation	Correlation Coefficient R^2^ PPO—Mal d 1	Correlation Coefficient R^2^ TPC—Mal d 1	Correlation Coefficient R^2^ TEAC—Mal d 1	Correlation Coefficient R^2^ ORAC—Mal d 1
All	−0.1164	0.0582	−0.1676	−0.0211
All control samples	−0.1635	−0.0115	−0.3207	−0.0375
All biofortified samples	−0.1524	0.1378	0.0006	−0.1382
‘Fiesta’ 2017	0.1463	0.0529	−0.1343	−0.4863
‘Jonica’ 2017	−0.7158 *	−0.4915	−0.3110	−0.3962
‘Golden Delicious’ 2017	−0.5614	−0.2115	−0.3889	0.1618
‘Jonagold’ 2017	−0.0444	0.1980	−0.4260	0.6741 *
‘Boskoop’ 2018	0.8589 **	−0.2949	−0.4697	0.0013
‘Jonica’ 2018	0.3496	−0.0322	0.0759	0.0767
‘Golden Delicious’ 2018	0.3847	0.5139	0.8740 *	0.3760
‘Jonagold’ 2018	−0.0296	−0.6023	−0.5536	0.4483
‘Elstar’ 2019	−0.4324	0.3780	0.4998	−0.2930
‘Fiesta’ 2017 Control	0.3081	−0.6859	−0.6037	−0.4795
‘Fiesta’ 2017 Selenium	−0.1074	0.5634	0.0820	−0.9576 ***
‘Jonica’ 2017 Control	−0.9364	−0.4338	−0.3799	−0.3814
‘Jonica’ 2017 Selenium	−0.5521	−0.4679	−0.2717	0.1818
‘Golden Delicious’ 2017 Control	0.5215	−0.4503	−0.4439	−0.5066
‘Golden Delicious’ 2017 Selenium	−0.4871	−0.7232 *	−0.5940	0.1390
‘Jonagold’ 2017 Control	−0.1373	0.7581	0.8501	−0.6730
‘Jonagold’ 2017 Selenium	0.7316 *	0.1126	0.5074	0.7491 *
‘Boskoop’ 2018 Control	0.7455	−0.7328	−0.8318	−0.3367
‘Boskoop’ 2018 Selenium	0.2508	−0.1612	−0.2445	0.7092
‘Jonica’ 2018 Control	0.0281	−0.6060	−0.4973	−0.6569
‘Jonica’ 2018 Selenium	0.8033	0.7256	0.7631	0.4636
‘Golden Delicious’ 2018 Control	−0.5166	0.9170	0.7411	0.3569
‘Golden Delicious’ 2018 Selenium	−0.8993	0.4206	0.8222	−0.9424
‘Jonagold’ 2018 Control	0.6978	−0.9217	−0.8754	−0.4095
‘Jonagold’ 2018 Selenium	−0.9821 *	−0.7753	−0.6820	0.6942
‘Elstar’ 2019 Control	−0.2390	0.1797	0.1275	−0.1005
‘Elstar’ 2019 Selenium	−0.4857	0.3917	0.5802	−0.3532

* *p* ≤ 0.05; ** *p* ≤ 0.01; *** *p* ≤ 0.001.

**Table 3:** Correlation coefficients between the content of Mal d 1 and the different phenolic compounds.

Cultivar and Year of Cultivation	Mal d 1	Mal d 1	Mal d 1	Mal d 1	Mal d 1	Mal d 1
	Chlorogenic Acid	Epicatechin	Procyanidin Trimers	Caffeoyl Glucosides	Σ Phloretin Glucosides	Σ Quercetin Glycosides
All 2017	−0.0379	0.2277	0.5165 ***	−0.2685	−0.0361	−0.1151
All controls 2017	−0.3064	0.1077	0.4866	−0.3484	0.3345	0.3230
‘Fiesta’ Control 2017	−0.9558 *	0.7474	0.9904 **	0.6394	−0.4251	−0.8979
‘Jonica’ Control 2017	0.4068	0.2401	−0.9204	0.0662	0.7636	−0.6081
‘Golden Delicious’ Control 2017	−0.5851	−0.8429	−0.6738	−0.2393	−0.0145	−0.7521
‘Jonagold’ Control 2017	−0.8553	0.5818	0.6812	−0.3038	0.6768	0.6710
All biofortified 2017	0.2869	0.3325	0.4929 **	−0.1586	−0.2357	−0.1782
‘Fiesta’ Selenium 2017	0.2344	0.8735 *	0.4806	0.1037	−0.6540	−0.6743
‘Jonica’ Selenium 2017	−0.0262	0.0871	0.6946	−0.6670	−0.2565	−0.0444
‘Golden Delicious’ Selenium 2017	0.6621	0.5236	0.6252	0.6760	0.8206 *	0.7218 *
‘Jonagold’ Selenium 2017	0.3544	−0.0528	0.4626	−0.0715	−0.0557	0.2322

* *p* ≤ 0.05; ** *p* ≤ 0.01; *** *p* ≤ 0.001.

## Data Availability

The data presented in this study are available on request from the corresponding author.

## Supplementary Materials

The following are available online, Table S1: Results of the determination of the selenium content, Mal d 1 content, polyphenol oxidase activity, total phenolic content, and antioxidant activity in all apple samples. Table S2: Results of the determination of phenolic compounds using HPLC-MS^n^.

## Sample Availability

Samples of the compounds are available from the authors.
