# Supplementary material for: Relationship between Phenolic Compounds, Antioxidant Properties, and the Allergenic Protein Mal d 1 in Different Selenium-Biofortified Apple Cultivars (Malus domestica)
Source: Molecules. 2021 Apr 30;26(9):2647. doi: 10.3390/molecules26092647 (PMC8124677; doi:10.3390/molecules26092647)
Supplement: Supplementary file 1 [file molecules-26-02647-s001.zip › molecules-1195936-supplementary.pdf]

Supplementary Material

Table S1. Results of the determination of the selenium content, Mal d 1 content, polyphenol oxidase activity, total phenolic content, and antioxidant activity in all apple samples. Data are given as mean value ± standard deviation (n = 4; n = 2 for Mal d 1).

Variety and Year of Cultivation	Application*	Se [µg/100 g f.w.]	Mal d 1 [µg/100 g d. w.]	PPO [Units/ 100 g f. w.]	TPC [mg GAE/ 100 g d. w.]	TEAC [mmol TE/ 100 g d. w.]	ORAC [mmol TE/ 100 g d. w.]
'Fiesta' 2017	control (HS)	0.1 ± 0.1	51.0 ± 19.2	10.4 ± 7.6	1,141.3 ± 419.7	15.3 ± 4.9	13.4 ± 5.4
	0.1 kg selenite (HS)	3.1 ± 1.5	68.8 ± 18.7	1.9 ± 0.3	838.5 ± 273.0	11.1 ± 2.6	4.6 ± 1.8
	0.1 kg selenate (HS)	3.1 ± 1.4	24.8 ± 4.8	2.5 ± 1.4	843.9 ± 54.5	13.0 ± 0.6	9.5 ± 0.5
'Jonica' 2017	control (HS)	0.7 ± 0.2	43.1 ± 4.5	3.2 ± 1.7	735.6 ± 66.6	5.5 ± 0.5	11.2 ± 1.4
	0.15 kg selenite (HS)	13.9 ± 1.3	37.3 ± 8.9	5.5 ± 3.0	843.0 ± 169.2	5.7 ± 0.9	14.1 ± 1.2
'Golden Delicious' 2017	control (HS)	0.4 ± 0.2	44.5 ± 3.2	12.0 ± 2.2	863.8 ± 123.8	6.8 ± 1.1	5.4 ± 1.6
	0.15 kg selenite (HS)	5.6 ± 0.5	36.5 ± 3.9	18.5 ± 2.8	797.9 ± 44.7	6.0 ± 0.3	1.9 ± 1.1
	0.15 kg selenate (HS)	5.6 ± 0.8	35.0 ± 3.2	24.5 ± 9.5	851.4 ± 14.9	7.6 ± 1.6	6.0 ± 1.4
'Jonagold' 2017	control (HS)	0.4 ± 0.2	107.5 ± 4.9	2.4 ± 0.7	938.2 ± 78.0	7.4 ± 0.9	11.0 ± 2.2
	0.15 kg selenite (HS)	5.6 ± 1.2	24.1 ± 4.2	4.1 ± 1.5	956.5 ± 36.7	11.0 ± 3.9	7.2 ± 1.2
	0.15 kg selenate (HS)	4.5 ± 1.6	53.3 ± 19.0	5.8 ± 5.2	893.8 ± 74.0	13.3 ± 1.3	9.5 ± 1.3
'Golden Delicious' 2018	control (BS)	0.3 ± 0.0	40.3 ± 5.7	142.3 ± 34.9	1,212.2 ± 164.3	12.1 ± 0.8	14.2 ± 1.5
	0.075 kg selenate (BS)	3.7 ± 0.4	29.9 ± 4.0	61.1 ± 17.5	1,206.9 ± 226.4	10.7 ± 1.1	8.8 ± 3.0
'Jonagold' 2018	control (BS)	0.2 ± 0.0	25.9 ± 3.5	33.5 ± 6.9	900.1 ± 136.4	11.2 ± 2.8	8.1 ± 1.2
	0.075 kg selenate (BS)	2.1 ± 0.7	39.0 ± 5.7	42.4 ± 35.0	815.4 ± 103.5	11.4 ± 0.8	15.5 ± 1.2
'Boskoop' 2018	control (OS)	0.4 ± 0.1	43.1 ± 6.5	41.8 ± 10.5	745.2 ± 66.1	15.6 ± 2.4	17.0 ± 7.1
	0.075 kg selenate (OS)	5.3 ± 0.3	30.9 ± 0.1	18.8 ± 4.7	743.5 ± 51.3	16.5 3.4	14.7 ± 0.9
'Jonica' 2018	control (OS)	0.3 ± 0.1	38.0 ± 7.7	11.4 ± 9.2	785.1 ± 177.3	12.8 ± 1.8	8.9 ± 1.5
	0.075 kg selenate (OS)	3.9 ± 0.7	32.6 ± 5.2	3.8 ± 1.5	841.7 ± 106.7	11.8 ± 1.6	5.6 ± 0.9
'Elstar' 2019	control (OS)	0.6 ± 0.0	46.9 ± 1.0	3.8 ± 0.8	828.1 ± 48.0	7.0 ± 0.3	14.1 ± 1.0
	0.15 kg selenate (OS)	8.7 ± 1.4	39.0 ± 3.3	35.5 ± 8.9	750.7 ± 121.4	5.7 ± 0.5	14.4 ± 0.5
	0.45 kg selenate (OS)	23.2 ± 2.7	53.4 ± 13.6	3.6 ± 1.1	902.7 ± 26.8	7.0 ± 0.2	12.3 ± 1.0

** Foliar spray rate of selenium per hectare and meter canopy height applied with a hand-held sprayer (HS), a backpack sprayer (BS) or a trailed orchard sprayer (OS).*

Table S2. Results of the determination of phenolic compounds using HPLC-MS^n. Data are in average ± standard deviation. The total phenolic content in mg/100 g d. w. was calculated by the sum of all quantitative determined phenolic compounds. For the four main phenolic compounds the content in mg/100 g d. w. and the percentage share is given (n = 4). Phloretin glycosides and Quercetin glycosides were summed up, respectively.

Variety and Year of Cultivation	Application*	Σ [mg/100 g d.w.]	Chlorogenic acid [mg/100 g d.w.]	%	Epicatechin [mg/100 g d.w.]	%	Procyanidin Trimer [mg/100 g d.w.]	%	Caffeeoyl-glucoside [mg/100 g d.w.]	%	Σ Phloretin glucosides	%	Σ Quercetin glycosides	%
'Fiesta' 2017	control (HS)	166.18 ± 48.57	66.43 ± 19.90	39.9	22.47 ± 2.28	14.7	15.51 ± 0.49	10.0	5.16 ± 0.26	3.3	13.01 ± 1.66	8.2	42.18 ± 31.01	22.9
	0.1 kg selenite (HS)	118.72 ± 23.16	50.13 ± 15.05	41.5	14.00 ± 4.38	11.6	11.81 ± 5.65	9.6	5.09 ± 0.11	4.4	9.28 ± 1.14	8.1	26.98 ± 1.77	23.5
	0.1 kg selenate (HS)	139.57 ± 11.16	56.83 ± 2.78	40.8	11.00 ± 0.57	7.9	12.35 ± 1.24	8.9	5.06 ± 0.28	3.6	12.24 ± 1.87	8.7	40.72 ± 7.41	29.0
'Jonica' 2017	control (HS)	77.77 ± 3.37	20.64 ± 2.58	26.5	7.12 ± 1.95	9.1	8.63 ± 0.64	11.1	5.23 ± 0.25	6.7	10.92 ± 0.61	14.0	23.81 ± 1.63	30.7
	0.15 kg selenite (HS)	96.94 ± 24.87	20.75 ± 2.85	21.9	7.11 ± 2.53	7.2	10.23 ± 1.12	10.9	5.24 ± 0.20	5.7	14.47 ± 5.13	14.7	37.72 ± 13.76	38.1
'Golden Delicious' 2017	control (HS)	123.14 ± 16.54	33.65 ± 3.92	27.4	11.86 ± 2.32	9.6	9.34 ± 2.33	7.5	14.81 ± 0.41	12.2	17.00 ± 5.37	14.1	35.09 ± 10.44	28.1
	0.15 kg selenite (HS)	144.19 ± 23.99	33.48 ± 0.29	23.7	14.98 ± 1.63	10.5	10.27 ± 1.29	7.2	13.88 ± 1.03	9.9	16.86 ± 2.92	11.7	53.33 ± 18.66	36.1
	0.15 kg selenate (HS)	127.35 ± 17.63	32.59 ± 2.78	25.8	12.89 ± 2.32	10.1	8.46 ± 1.67	6.6	13.42 ± 1.64	10.6	16.04 ± 1.65	12.6	42.57 ± 9.68	33.2
'Jonagold' 2017	control (HS)	137.76 ± 20.98	28.36 ± 3.77	21.2	13.19 ± 2.40	9.6	14.42 ± 2.40	10.4	4.99 ± 0.12	3.7	16.74 ± 3.34	12.1	58.69 ± 16.61	42.0
	0.15 kg selenite (HS)	131.90 ± 16.66	30.20 ± 5.48	23.4	14.75 ± 3.46	11.2	11.45 ± 1.24	8.7	8.28 ± 3.79	6.3	19.63 ± 6.25	14.7	46.20 ± 12.20	34.7
	0.15 kg selenate (HS)	134.09 ± 6.74	31.25 ± 3.09	23.3	13.04 ± 2.37	9.7	11.14 ± 1.89	8.3	8.90 ± 4.57	6.7	18.96 ± 0.29	14.2	49.37 ± 6.07	36.8

** Foliar spray rate of selenium per hectare and meter canopy height applied with a hand-held sprayer (HS)*
